# A Probiotic Mixture Regulates T Cell Balance and Reduces Atopic Dermatitis Symptoms in Mice

**DOI:** 10.3389/fmicb.2018.02414

**Published:** 2018-10-15

**Authors:** Han Wool Kim, Rira Hong, Eun Young Choi, KeeSun Yu, Narae Kim, Jin Yi Hyeon, Kwang Keun Cho, In Soon Choi, Cheol-Heui Yun

**Affiliations:** ^1^Department of Agricultural Biotechnology, Research Institute for Agriculture and Life Sciences, Seoul National University, Seoul, South Korea; ^2^Department of Biological Science, College of Medical and Life Sciences, Silla University, Busan, South Korea; ^3^Department of Animal Resources Technology, Gyeongnam National University of Science and Technology, Jinju, South Korea; ^4^Institute of Green Bio Science and Technology, Seoul National University, Pyeongchang, South Korea

**Keywords:** probiotics, atopic dermatitis, dendritic cell, T cell balances, health food

## Abstract

Atopic dermatitis (AD) is a chronic inflammatory skin disorder with a complex etiology involving the immune response. Recent studies have demonstrated the role of certain probiotics in the treatment and prevention of AD. However, the mechanism by which these probiotics regulate the immune system remains unclear. In this study, we examined the immunomodulatory capacity of Duolac ATP, a mixed formulation of probiotics, both *in vitro* and *in vivo*. Results showed that the expression of programmed death-ligand 1(PD-L1) was significantly upregulated on bone marrow-derived dendritic cells (BMDCs) treated with Duolac ATP. Furthermore, the anti-inflammatory cytokines IL-10 and TGF-beta were both upregulated when BMDCs were treated with Duolac ATP. The percentage of proliferated regulatory T cells (Tregs) was enhanced when CD4^+^ T cells were co-cultured with Duolac ATP-treated BMDCs on plates coated with anti-CD3/CD28 antibodies. Intriguingly, IL-10 secretion from CD4^+^ T cells was also observed. The AD symptoms, histologic scores, and serum IgE levels in AD mice were significantly decreased after oral treatment with Duolac ATP. Moreover, the Th1-mediated response in AD-induced mice treated with oral Duolac ATP showed upregulation of IL-2 and IFN-gamma as well as of downstream signaling molecules T-bet, STAT-1, and STAT-4. Conversely, Duolac ATP suppressed Th2 and Th17 responses in AD-like mice, as evidenced by the downregulation of GATA-3, C-maf, IL-4, IL-5, and IL-17. Additionally, Duolac ATP increased the number of Tregs found at Peyer’s patches (PP) in treated AD mice. These results suggest that Duolac ATP modulates DCs to initiate both Th1 and Treg responses in AD mice. Thus, Duolac ATP represents a potential preventative agent against AD and could serve as an effective immunomodulator in AD patients.

## Introduction

Atopic dermatitis (AD) is a chronic inflammatory skin disorder that affects the structural and barrier functions of the skin. Major symptoms of AD include excessive pruritus and eczema. Although the exact pathophysiology of AD is not yet fully understood, it can be caused by a combination of genetic, environmental, allergic, and microbial factors (Abramovits, 2005). Like other allergic diseases, AD is caused by an immune response – primarily T-helper (Th) 2 cell hypersensitivity. A balance between Th1 and Th2 cells is important for disease induction and tolerance. In AD patients, overexpression of Th2 cytokines, including interleukin (IL)-4, IL-5, and IL-13, upregulate IgE production, resulted in eosinophil accumulation within the dermis (Woodfolk, 2007; Hong et al., 2017). Although steroid drugs are effective therapies, they have serious side effects, including dermal atrophy, acne, cataracts, growth retardation, and skin irritation. As such, long-term steroid treatment should generally be avoided (Gupta and Chow, 2003; Hengge et al., 2006). Other alternative therapeutic agents are being investigated, including herbs, phytochemicals, vitamins, and probiotics (Mainardi et al., 2009).

Probiotics, which are non-invasive, non-pathogenic, Gram-positive bacteria with known health-promoting effects, are primarily found in fermented food and feed products (Mercenier et al., 2003; Rather et al., 2016). An adequate quantity of probiotics can be helpful for host gut homeostasis, which is achieved through immune system modulation and the production of antimicrobial agents that block the adhesion of pathogens and their toxins (Reid et al., 2003). Recent studies have reported that probiotics are also effective in preventing allergic disorders in mice and humans (Isolauri et al., 2000; Isolauri, 2001; Cuello-Garcia et al., 2015). *Lactobacillus plantarum* supplementation reduced the scoring atopic dermatitis (SCORAD) index in young atopic patients (Isolauri, 2001; Prakoeswa et al., 2017). In another study, *Lactobacillus casei* supplementation prevented the development of AD in NC/Ng mice (Tanaka et al., 2009). However, the mechanism by which probiotics function is still not completely understood.

Dendritic cells (DCs) are antigen-presenting cells that can effectively induce a primary immune response to pathogens as well as maintain tolerance to self-antigens (Banchereau and Steinman, 1998). DCs play a key role in bridging innate and adaptive immune responses (Banchereau and Steinman, 1998; Drakes et al., 2004; Lee and Iwasaki, 2007). Depending on the stimulus, DCs can secrete cytokines and induce naïve T cell differentiation toward Th1, Th2, Th17, or Treg lineages. Therefore, much attention has focused on the impact of DC priming by probiotics to modulate T cell responses (Powrie, 2004). Some probiotic strains, including *Lactobacillus* and *Bifidobacterium*, modulate the action of DCs to produce IL-10 and IL-12 along with the expression of co-stimulatory molecules (Christensen et al., 2002; Hart et al., 2004). Several *Lactobacillus* strains have been shown to inhibit T cell proliferation, induce IL-10 and TGF-beta production, and modify Th1 and Th2 cytokine production in various models of autoimmune diseases (Lavasani et al., 2010). In another study, *Bifidobacterium lactis* inhibited TGF-beta production (Lindfors et al., 2008). These findings suggest that probiotics should be carefully selected so that the resultant immune response is appropriate for the desired clinical application.

Duolac ATP is a probiotic preparation containing four probiotics strains: *L. casei*, *L. plantarum*, *Lactobacillus rhamnosus*, and *B. lactis*. Duolac ATP has been previously evaluated for anti-inflammatory activity in a trinitrobenzene sulfonic (TNBS)-induced colitis model (Cha et al., 2014) and a 1-chloro-2,4-dinitrobenzene (DNCB)-induced AD model (Kim et al., 2016). However, these studies focused on the therapeutic effects of Duolac ATP through fragmentary indicators.

The aim of our study was to evaluate the efficacy of Duolac ATP within innate immune systems via bone marrow-derived dendritic cells (BMDCs) and to use a transcription factor and cytokine analysis of atopic mice to explore the mechanism by which Duolac ATP overcomes AD.

## Materials and Methods

### Animal

Female, 7 to 10 week old, Balb/c from Orient (Gapyeong, South Korea), or 4 week old NC/Nga mice from the Shizuoka Laboratory Animal Center (Tokyo, Japan) were purchased. The mice were randomized and housed in stainless steel cages in a controlled environment with a 12 h light-dark cycle. All the experimental procedures were carried out in accordance with the Animal Use and Care Protocol approved by the Institutional Animal Care and Use Committee (IACUC) at Seoul National University, Seoul, Korea (Approval No. SNU-170428-1).

### Probiotics

Probiotic strains were obtained from Cell Biotech Co., Ltd. (Gimpo, Korea) as a powder form, containing 1 × 10^11^ CFU/g. In this study, we used Duolac ATP that is composed of four different strains of probiotics: *L. casei* CBT LC5 (KCTC12398BP), *L. plantarum* CBT LP3 (KCTC10782BP), *L. rhamnosus* CBT LR5 (KCTC12202BP), and *B. lactis* CBT BL3 (KCTC11904BP).

### Generation and Culture of BMDCs *in vitro*

Bone marrow (BM) cells were isolated from femurs of mice. Red blood cells were depleted using RBC-lysis buffer (Sigma-Aldrich, MO, United States) and BM cells were cultured in a complete RPMI with 20 ng/ml GM-CSF (Creagene, South Korea). The complete RPMI was composed of RPMI-1640 supplemented with 10% fetal bovine serum, 20 mM HEPES, 1 mM sodium pyruvate, 220 nM 2-Mercaptoethanol, 100 μg/ml Gentamicin (all from Sigma-Aldrich). At day 0, BM cells were seeded at 3 × 10^6^ cells/well in 6-well plate in 3 ml media, and 2 ml of fresh media was added at day 3. At day 5, a half of the culture supernatant was discarded, and 3 ml of fresh media was added. At day 7, suspended BM cells were harvested and sorted by CD11c MicroBeads UltraPure kit (Miltenyi Biotec Inc., CA, United States). Suspended CD11c^+^ BM cells, i.e., BMDCs. BMDCs were seeded at 2 × 10^5^ cells/well in 96-well plate and stimulated with Duolac ATP or 100 ng/ml lipopolysaccharide (LPS) in a complete RPMI. After the incubation for 24 h, the supernatant was collected for cytokine concentration.

### *In vitro* CD4^+^ T Cell Stimulation

CD4^+^ T cells was isolated from mesenteric lymph node (mLN) from wild type mice using mouse CD4 T lymphocyte enrichment Set (BD Biosciences, CA, United States). CD4^+^ T cells were labeled with CellTrace^TM^ Violet (CTV) Cell Proliferation Kit (Thermo Fisher Scientific, Rockford, IL, United States). CD4^+^ T cells (2 × 10^5^ cells/well) were co-cultured with Duolac-treated BMDCs (2 × 10^4^ cells/well) incubated on anti-CD3/CD28 mAbs (BD Biosciences)-coated 96-well plate. After the incubation for 72 h, the cells were examined for proliferation of Foxp3^+^CD4^+^ T cells and the supernatant was collected and examined for IL-10 and IFN-gamma concentration.

### Mouse AD Model

The back of NC/Nga mice was shaved and dorsal skin and ears were sensitized with house dust mite (HDM) extracts (Biostir, Japan) or DNCB (Sigma-Aldrich) twice a week for 3 weeks to induce AD-like skin lesions. After following the last treatment, mice were administered with PBS (200 μL/day) or Duolac ATP (2 × 10^9^ CFU/200 μL/day) every day or three times a week for 28 days (**Supplementary Figure S1**). At the end of the treatment, the mice were anesthetized with CO_2_. Blood samples were collected by heart puncture into heparinized tubes. The sera were then collected by centrifugation for 10 min at 3,000 rpm and stored at −80°C, until further use for ELISA. The mice, at the end of experiment, were sacrificed, and dorsal skin and ear samples were collected for histological analysis and TUNEL assay, respectively. Peripheral blood mononuclear cells (PBMCs), purified from blood of mice treated with/without Duolac ATP, were purified by density gradient centrifugation using Histopaque^®^-1077 (Sigma-Aldrich), and stored at −80°C, until further use for qPCR and western blot. Mesenteric lymph node (mLN) and Peyer’s patches (PP) were taken and the distribution of immune cells was measured by flow cytometric analysis.

### Histology

The dorsal skin was removed and fixed in a 4% paraformaldehyde (Sigma-Aldrich, MO, United States). The paraffin-embedded skin sections were heat immobilized, deparaffinized by immersing in xylene (Sigma-Aldrich), rehydrated using a graded series of ethanol, and washed with distilled water. The dorsal skin samples were then cut and subjected to hematoxylin and eosin (H&E) (Sigma-Aldrich) staining. Samples were then examined under the light microscopy (Leica Microsystems, Wetzlar, Germany) for histological evaluation. All clinical and histological evaluations were performed in a blinded manner.

### TUNEL Assay

To visualize DNA fragmentation, a marker of apoptosis, TUNEL staining was performed using an In Situ Cell Death Detection Kit (Roche, Mannheim, Germany) according to the manufacturer’s protocol. The sections were post-fixed with ethanol-acetic acid (2:1) and rinsed. The sections were then incubated with proteinase K (100 mg/mL), rinsed, and incubated in 3% H_2_O_2_, permeabilized with 0.5% Triton X-100, rinsed again, and incubated in the TUNEL reaction mixture. The sections were rinsed and visualized using Converter-POD with 0.03% 3,30-diaminobenzidine (DAB). Then, the sections counterstained with eosin were examined for TUNEL staining by using an optical microscope (Olympus BX53, Japan).

### RNA Isolation and qPCR

Total RNA was isolated from PBMCs by TRIzol^®^ reagent (Life Technologies, Carlsbad, CA, United States). One microgram of RNA was reverse-transcribed in a 20 μl reaction containing random primers (500 μg/ml), dNTP (10 mM), 5× first strand buffer, DTT (0.1 M), Superscript III enzyme (200 U/μl) and RNase inhibitor (10 U/μl) (all from Invitrogen, Carlsbad, CA, United States). RNA was reverse transcribed with HyperScript reverse transcription reagents (GeneAll Biotechnology, Seoul, Korea), and quantitative PCR (qPCR) was performed with the SYBR Green Supermix (iQ SYBR Green Supermix, Bio-Rad Laboratories, Hercules, CA, United States) on the LightCycler 480 Real-Time PCR System (Roche, Indianapolis, IN, United States). This was then used to calculate the relative amounts of target mRNA in test samples. Quantities of all targets in test samples were normalized to the corresponding GAPDH levels. Primers for IL-2 (forward : 5′-CCT GAG CAG GAT GGA GAA TTA CA-3′, reverse: 5′-TCC AGA ACA TGC CGC AGA G-3′), IL-4 (forward: 5′-ACA GGA GAA GGG ACG CCA T-3′, reverse: 5′-GAA GCC CTA CAG ACG TCA-3 ′), IL-5 (forward: 5′-GGG CTT CCT GCT CCT ATC TA-3′, reverse: 5′-CAG TCA TGG CAC AGT CTG AT-3′), IL-10 (forward: 5′-CAA CAT ACT GCT AAC CGA CTC CT-3′, reverse: 5′-TGA GGG TCT TCA GCT TCT CAC-3′), IL-17 (forward: 5′-TCT GAT GCT GTT GCT GCT G-3′, reverse: 5′-ACG GTT AGA GGT AGT CTG AGG-3′), IFN-gamma (forward: 5′- CAG CAA CAA CAT AAG CGT CA-3′, reverse: 5′-CCT CAA ACT TGG CAA TAC TCA-3′), TGF-beta (forward: 5′- GTG TGG AGC AAC ATG TGG AAC TCT-3′, reverse: 5′-TTG GTT CAG CCA CTG CCG TA-3′), GAPDH (forward: 5′-CAT GGC CTT CCG TGT TCC TA-3′, reverse: 5′-CCT GCT TCA CCA CCT TCT TGA T-3′) were synthesized from Bioneer Inc (Daejeon, Korea).

### Western Blot

Total protein was isolated from PBMCs by using RIPA buffer (Abcam, Cambridge, United Kingdom). The amount of proteins was quantified by BCA Protein assay kit (Thermo Fisher Scientific, Rockford, IL, United States), with bovine serum albumin (BSA) as a standard. Each protein sample was loaded onto 10% SDS-polyacrylamide gel and transferred to nitrocellulose membrane (Schleicher & Schuell BioScience, Germany) for 90 min at 4°C, and blocked with 5% skim milk in TBST (1 M Tris–HCl, 5 M NaCl, 10% Tween-20) for 1 h at room temperature. The blots were incubated with anti-GATA3, -T-bet, -C-maf, -STAT1, -P-STAT1, -STAT4, -P-STAT4 or -beta-actin (all from Abcam) antibodies overnight at 4°C. Primary antibody-bound membranes were incubated with goat anti-rabbit IgG-HRP antibody (Santa Cruz Biotechnology, United States) for 1 h at room temperature. The target protein was visualized with enhanced chemiluminescence system (GE Healthcare Life Sciences, United States), followed by analysis using ChemiDoc XRS (Bio-Rad).

### Enzyme-Linked Immunosorbent Assay (ELISA)

The immunological response of the mice following HDM extract or DNCB-induced AD was monitored by measuring the serum levels of mouse IL-10, IL-12p40, TGF-beta, IFN-gamma (all from R&D Systems, United States) and IgE (BD Biosciences). Mouse TGF-beta, IL-10 and IL-12p40 were measured from the supernatants taken after the BMDC cultured with Duolac ATP by using ELISA DuoSet kits (all from R&D Systems). Mouse IFN-gamma was measured from supernatants taken after the CD4^+^ T cells treated with BMDCs supernatants by using ELISA DuoSet kit (R&D Systems). Briefly, 96-well microplate (Nunc) was pre-coated with 100 μl/well of capture antibody. After blocking with 1% BSA for 1 h at room temperature, 100 μl/well of supernatant along with the standard solution diluted in diluent buffer was added and incubated for 2 h at room temperature. After the wash PBS for three times, 100 μl/well of detection antibody was added and incubated for 2 h at room temperature, followed by addition of the Streptavidin-HRP in PBS. After the incubation for 20 min at room temperature, tetramethylbenzidine (TMB, Millipore) was added to develop the color and then the reaction was stopped by adding 50 μl of 2 M H_2_SO_4_. The absorbance at wavelength 450 nm was measured by a microplate reader (Molecular Devices).

### Phenotypic and Functional Examination of Immune Cells by Using Flow Cytometry Analysis

In order to examine activation status of the cells, BMDCs were treated with Duolac ATP or LPS for 24 h at 37°C. The cells were stained with anti-mouse CD86-FITC, PD-L1-PE, MHC II-PE-cy7, CD11c-APC (all from BD Biosciences) for 20 min at 4°C in the dark. To test the increase of Treg, CTV labeled CD4^+^ T cells cultured with Duolac-treated BMDCs for 3 days were stained with anti-mouse CD4-PE (BD Biosciences). After surface staining, CD4^+^ T cells were fixed and stained with anti-mouse Foxp3-APC mAb (BioLegend, Dedham, MA, United States) using FOXP3 Fix/Perm Buffer Set (BioLegend). *In vivo* examination, single cells from mLNs and PP were isolated from AD mice. Population changes of DCs and Tregs were examined as aforementioned. To analyze for subpopulation of Th cells, total mLN and PP cells were stimulated with PMA and ionomycin (Sigma-Aldrich) in the presence of brefeldin A for 4 h. After the stimulation, the cells were stained with appropriate combination of anti-mouse CD11c-APC, CD4-bv605, Foxp3-APC, IFN-gamma-PE, IL-4-bv605, and IL-17-APC-cy7 mAb (all from Biolegend). The cells were washed and the expression was examined using a FACSCanto II (BD Biosciences). All flow cytometric data acquired were analyzed with FlowJo software (Tree Star, Ashland, OR, United States).

### Statistical Analysis

The levels of significance for comparison between samples were determined by Tukey’s multiple comparison test by using GraphPad InStat software (Ver 5.01, GraphPad). The data in the graphs were expressed as the mean ± SEM.

## Results

### Duolac ATP Effectively Induces Regulatory Immune Responses by BMDCs

**FIGURE 1:**
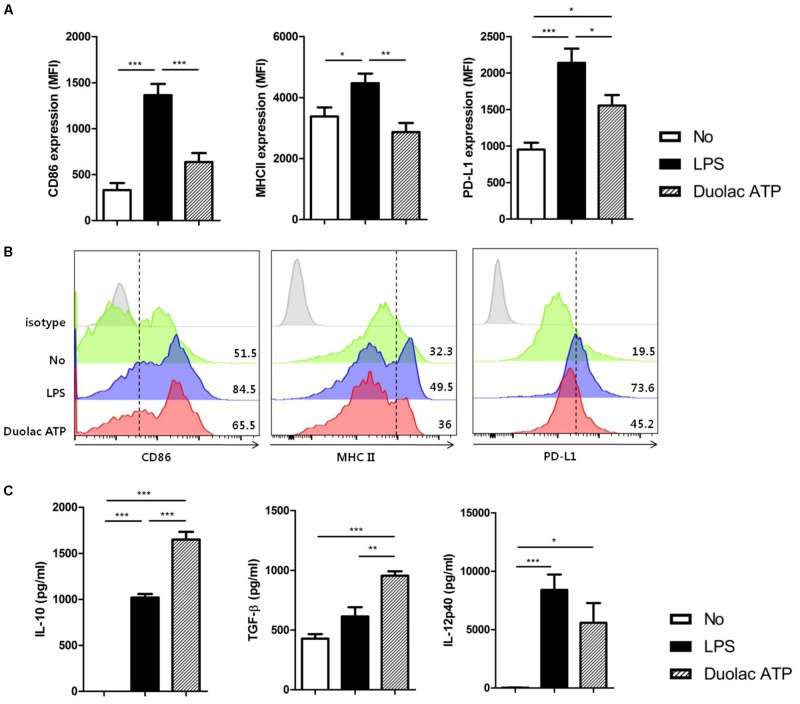
Duolac ATP induced regulatory molecules in bone marrow-derived dendritic cells (BMDCs). BMDCs were treated with LPS and/or 2 × 10^6^ CFU of Duolac ATP for 24 h. **(A)** The expression of surface markers and **(B)** a representative histogram of CD86, MHC II, and PD-L1 on BMDCs were measured by flow cytometry. **(C)** The expression of cytokines in the supernatants was measured by ELISA. Data are representative of at least three experiments. ^∗^*P* < 0.05, ^∗∗^*P* < 0.01, ^∗∗∗^*P* < 0.001 using one-way ANOVA with Tukey’s multiple comparison test. Bars indicate mean ± SEM.

We first examined the harmful effects, if any, of Duolac ATP on BMDCs. BMDCs were treated with various concentrations of Duolac ATP and their components. Regardless of the probiotic type, BMDC survival was significantly reduced when treated with over 2 × 10^6^ CFU (**Supplementary Figure S2**), suggesting that the optimal concentration is less than 2 × 10^6^ CFU. Based on these results, we decided to use a Duolac ATP concentration of 2 × 10^5^ CFU. BMDCs treated with Duolac ATP showed similar surface expressions of the co-stimulatory molecules CD86 and MHC II compared with untreated cells within the same intensity (**Figure 1A**) and percentage of cells (**Figure 1B** and **Supplementary Figure S3**). However, Duolac ATP-treated BMDCs demonstrated a significantly higher expression of PD-L1 compared with untreated cells within the same intensity (**Figure 1A**) and percentage of cells (**Figure 1B** and **Supplementary Figure S3**). Next, immunomodulatory cytokines were examined in the culture supernatant from BMDC treated with Duolac ATP. These results showed that Duolac ATP induced a significant amount of IL-10, an anti-inflammatory cytokine, at a rate 1.5-fold greater than cells treated with LPS (**Figure 1C**). TGF-beta production was also increased when BMDCs were treated with Duolac ATP, whereas IL-12p40 production was slightly lower than that of those treated with LPS (**Figure 1C**). Taken together, these results indicate that Duolac ATP effectively induced a regulatory immune response in BMDCs.

### BMDCs Treated With Duolac ATP Promote Proliferation of Tregs *in vitro*

**FIGURE 2:**
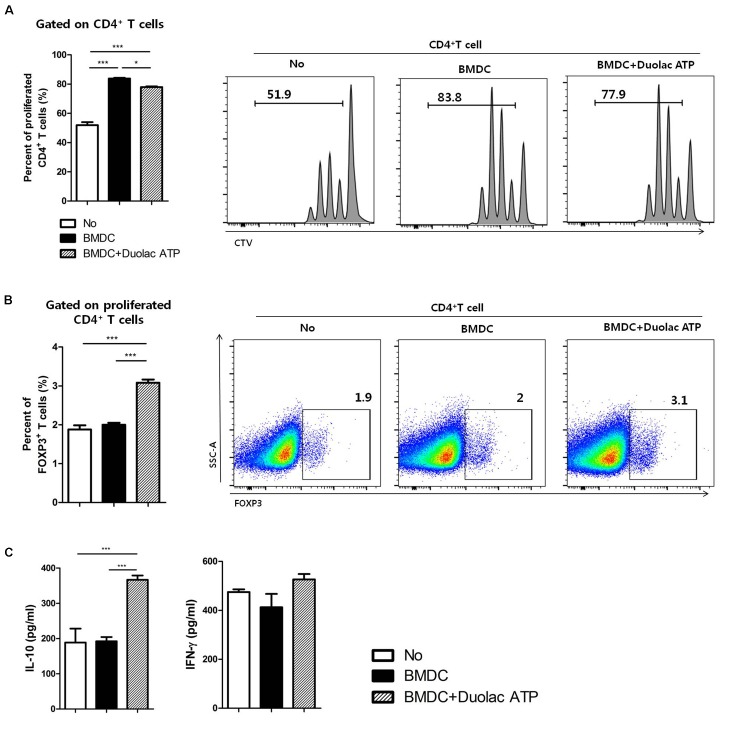
Bone marrow-derived dendritic cell treated with Duolac ATP promotes proliferation of Tregs *in vitro*. BMDCs treated with Duolac ATP were co-cultured with CD4^+^ T cells for 3 days on an anti-CD3/CD28 mAbs-coated plate. The percentage of the proliferated **(A)** total CD4^+^ T cells and **(B)** proportion of CD4^+^Foxp3^+^ T cells among CD4^+^ T cells were analyzed by flow cytometry. At the same time, supernatants were harvested and examined for **(C)** the production of IL-10 and IFN-gamma in the CD4^+^ T cells using ELISA. Data are representative of at least three experiments. ^∗^*P* < 0.05, ^∗∗∗^*P* < 0.001 using one-way ANOVA with Tukey’s multiple comparison test. Bars indicate mean ± SEM.

We investigated whether Duolac ATP could induce Treg differentiation. CD4^+^ T cells were co-cultured with Duolac ATP-treated BMDCs on an anti-CD3/CD28 mAbs-coated plate for 3 days. The results showed that BMDC-induced CD4^+^ T cell proliferation was higher than that in the control group, but it was slightly reduced when co-cultured with Duolac ATP-treated BMDCs (**Figure 2A**). The ratio of Foxp3^+^ Tregs in the proliferated CD4^+^ T cells was also significantly increased in the Duolac ATP-treated BMDC group compared to the group treated with BMDC alone or controls (**Figure 2B**). Immunomodulatory cytokines were then examined in the culture supernatant from CD4^+^ T cells co-cultured with Duolac ATP-treated BMDCs. IL-10 was increased in a similar fashion to Foxp3^+^ Treg proliferation. However, the expression of IFN-gamma was not affected by Duolac ATP treatment (**Figure 2C**). Taken together, these results indicate that Duolac ATP-treated BMDCs were able to induce Treg proliferation with a unique profile of cytokine induction.

### Amelioration of AD in Mice Treated With Duolac ATP

**FIGURE 3:**
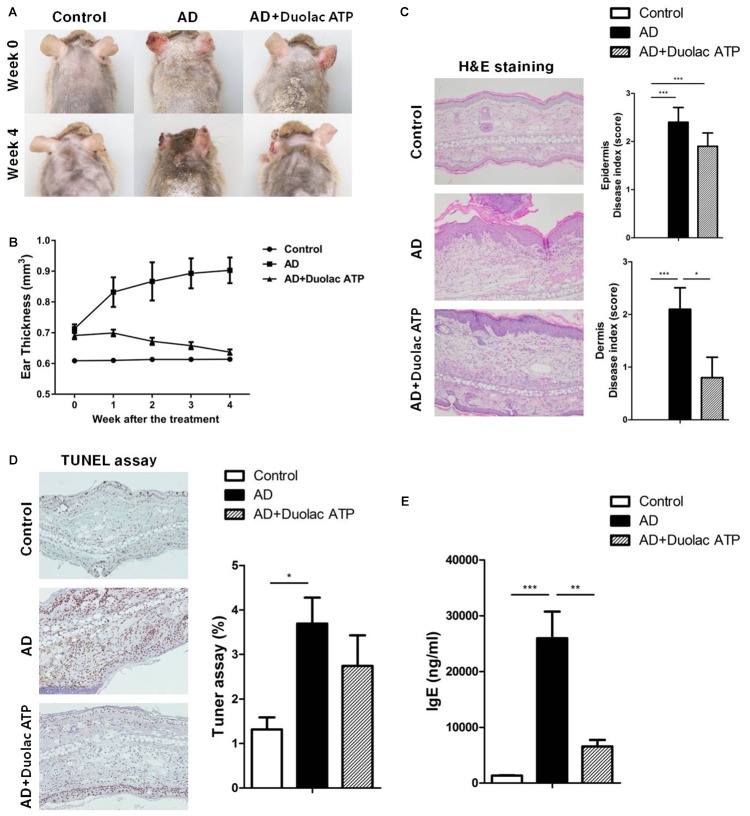
Amelioration of AD symptoms in mice treated with Duolac ATP. NC/Nga mice were sensitized by exposing them to HDM extract twice a week for 3 weeks. PBS or Duolac ATP was then orally administered for 4 weeks. **(A)** Atopic symptoms and **(B)** ear thickness were scored every week for the last 4 weeks. At the end of the experiment, **(C)** the results of the histological analysis of cell infiltration by H&E staining (left panel, one representative from 10 samples) and the epidermis and dermis index score (right panel) were examined. **(D)** TUNEL assay on dermis samples (left panel, one representative from 10 samples) and the percentage of the plot (right panel) are shown. **(E)** Blood samples were acquired, and serum IgE levels were measured by ELISA. ^∗^*P* < 0.05, ^∗∗^*P* < 0.01, ^∗∗∗^*P* < 0.001 using one-way ANOVA with Tukey’s multiple comparison test. Bars indicate mean ± SEM.

To determine the therapeutic properties of Duolac ATP *in vivo*, we established a mouse model for AD-like skin lesions. The NC/Nga mice were sensitized with HDM extracts twice a week for 3 weeks. The mice were then administered phosphate-buffered saline (PBS) (200 μL/day) or Duolac ATP (2 × 10^9^ CFU/day) for 4 weeks, as shown in **Supplementary Figure S1**. No significant changes were found in the mice’s physical habitus or spleen weight during the feeding period (**Supplementary Figure S4**). Compared to the control group, the AD group showed severe atopic symptoms, such as itching, erythema/hemorrhage, edema, excoriation/erosion, and scaling/dryness, at week 4 (**Figure 3A**). Interestingly, however, the atopic symptoms of AD mice treated with Duolac ATP were less severe. We also performed weekly examinations of ear thickness after the induction of AD. Compared to the control group, the ear thickness of the AD group increased in a time-dependent manner. However, the AD group treated with Duolac ATP showed an attenuated increase in ear thickness (**Figure 3B**). AD is a chronic inflammatory skin disease that not only causes the aforementioned clinical symptoms but also increases epidermal and dermal thickness via activation and infiltration of immune cells (Shin et al., 2016). To investigate the increase in dermal thickness, we stained the skin and measured the epidermis and dermis based on known disease indices. Epithelial hypertrophy and hyperkeratosis coincident with immune cell infiltration were observed in the AD group (**Figure 3C**, left panel). Furthermore, some mice showed epidermal collapse with bleeding and extensive cartilaginous ulceration. The AD group treated with Duolac ATP had fewer histopathologic anomalies (**Figure 3C** right panel). Furthermore, by conducting a TUNEL assay, we were able to examine the degree of apoptosis. In the AD group, several apoptotic cells were observed, whereas this was less obvious in the AD group treated with Duolac ATP (**Figure 3D**). Serum IgE levels were significantly increased in the AD group compared to the control group, whereas the AD group treated with Duolac ATP showed a significant decrease (**Figure 3E**). These results suggest that Duolac ATP efficiently ameliorates the symptoms of AD and decreases overall serum IgE levels.

### Maintenance of Systemic T Cell Balance in AD Mice Treated With Duolac ATP

**FIGURE 4:**
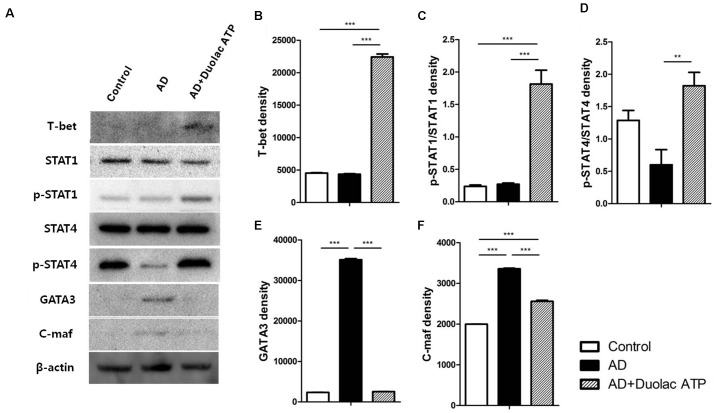
Expression changes on transcriptional factors involved in the maintenance of T cell balance in AD mice administered Duolac ATP. Nc/Nga mice (*n* = 6 per group) were sensitized by exposing them to HDM extracts twice a week for 3 weeks. They were then subjected to oral administration of Duolac ATP. PBMCs collected at week 4 were used to make lysates, which were used to examine the expression of transcriptional factors using Western blotting. **(A)** The expression of T-bet, STAT1, p-STAT1, STAT4, p-STAT4. GATA-3, C-maf, and beta-actin as an internal control are shown. Density was measured using a densitometer for semi-quantitation for **(B)** T-bet, **(C)** STAT1, **(D)** STAT4, **(E)** GATA 3, and **(F)** C-maf. ^∗∗^*P* < 0.01, ^∗∗∗^*P* < 0.001 using one-way ANOVA with Tukey’s multiple comparison test. Bars indicate mean ± SEM.

**FIGURE 5:**
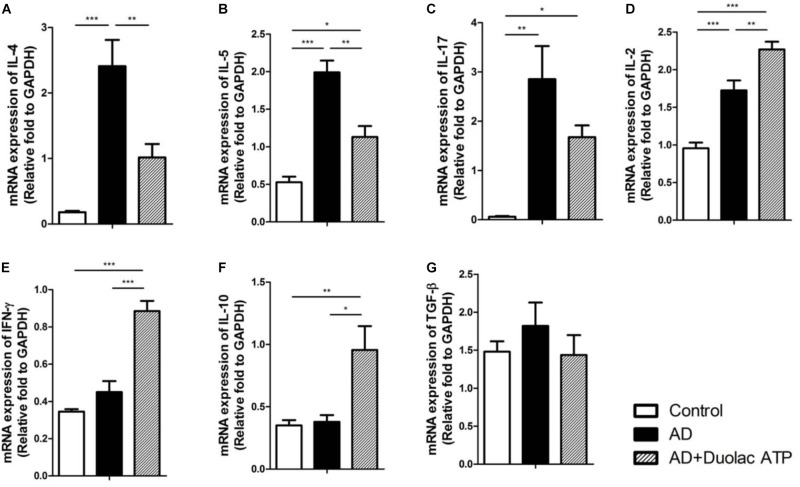
mRNA expression of cytokine levels from PBMCs in HDM-sensitized Nc/Nga mice treated with Duolac ATP. Nc/Nga mice (*n* = 6 per group) were sensitized by exposing them to HDM extracts six times over 3 weeks. They were then subjected to oral administration with Duolac ATP. PBMCs were collected at week 4, and RNA was extracted for cDNA synthesis. qPCR was performed to examine the mRNA expression of **(A)** IL-4, **(B)** IL-5, **(C)** IL-17, **(D)** IL-2, **(E)** IFN-γ, **(F)** IL-10, and **(G)** TGF-β. Relative fold changes of target genes were compared with that of the housekeeping gene, GAPDH. ^∗^*P* < 0.05, ^∗∗^*P* < 0.01, ^∗∗∗^*P* < 0.001 using one-way ANOVA with Tukey’s multiple comparison test. Bars indicate mean ± SEM.

We investigated the transcription factors related to Th1 and Th2 cell differentiation in AD mice treated with Duolac ATP. T-bet (**Figures 4A,B**), STAT-1 (**Figures 4A,C**), and STAT-4 (**Figures 4A,D**) are factors that induce Th1 cell differentiation; these were all expressed at a significantly higher rate in PBMCs from AD mice treated with Duolac ATP compared to untreated AD mice. It was further noted that Th2 differentiation factors GATA-3 (**Figures 4A,E**) and C-maf (**Figures 4A,F**) were increased in PBMCs from AD mice. These results suggested that Duolac ATP led to the balance between Th1 and Th2 cells in AD mice by preferentially increasing the release of Th1 differentiation factors. We also examined the mRNA expression of Th1 and Th2 cytokines in PBMCs from AD mice given Duolac ATP. We found higher rates of IL-4 (**Figure 5A**) and IL-5 (**Figure 5B**) expression in AD mice, as was expected from a typical Th2 response. Furthermore, IL-17 appeared to be increased in AD mice (**Figure 5C**). However, AD mice treated with Duolac ATP showed a decrease in Th2 and Th17, with a concurrent increase in IL-2 (**Figure 5D**) and IFN-gamma (**Figure 5E** and **Supplementary Figure S6A**). The mRNA expression of IL-10 was also increased in AD mice treated with Duolac ATP, whereas there was no change in TGF-beta (**Figures 5F,G**). These results indicate that Duolac ATP can induce Th1- but not Th2- or Th17-driven responses.

### Maintenance of Intestinal T Cell Balance in AD Mice Treated With Duolac ATP

**FIGURE 6:**
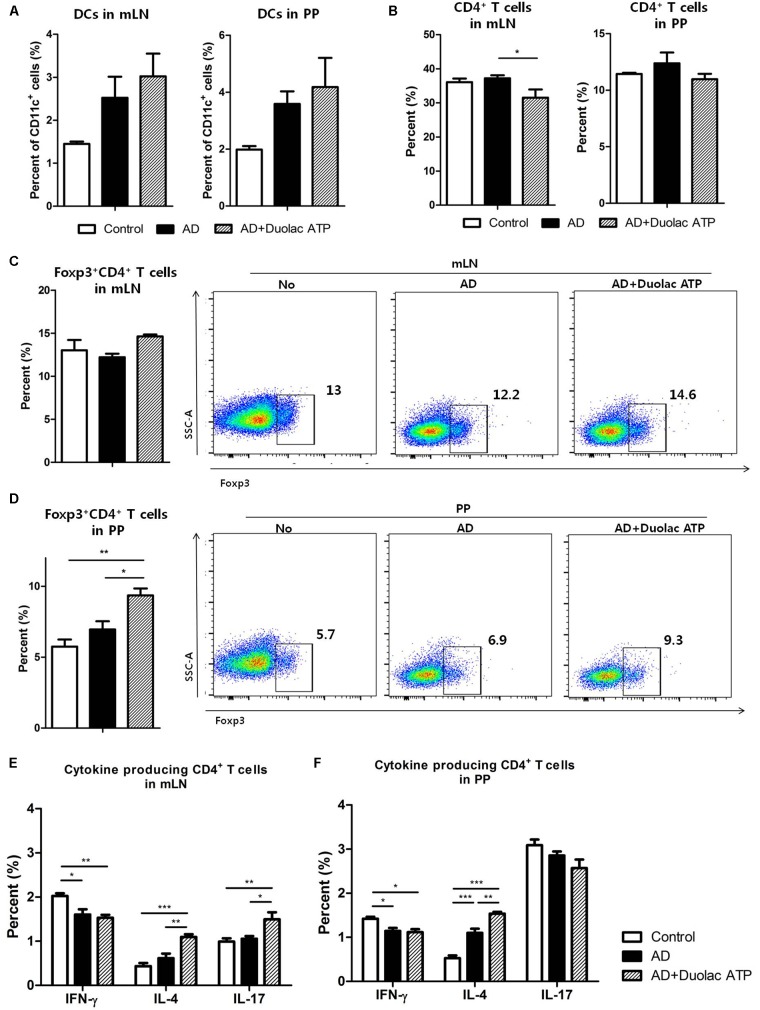
Composition of immune cells from mLN and PP in DNCB-sensitized Nc/Nga mice treated with Duolac ATP. Nc/Nga mice were sensitized by exposing them to DNCB twice a week for 3 weeks. They were then subjected to oral administration of Duolac ATP. Mesenteric lymph node (mLN) and Peyer’s patches (PP) collected at week 4 were used to make single cells, which were used to examine the composition of immune cells. The percentages of **(A)** DCs, **(B)** CD4^+^ T cells, and Foxp3^+^CD4^+^ T cells from **(C)** mLN and **(D)** PP were examined using flow cytometry. mLN and PP cells were stimulated with PMA/ionomycin in the presence of brefeldin A for 4 h. IFN-gamma, IL-4, and IL-17 producing CD4^+^ T cells from **(E)** mLN and **(F)** PP were examined after intracellular staining using flow cytometry. Data are representative of at least three experiments. ^∗^*P* < 0.05, ^∗∗^*P* < 0.01, ^∗∗∗^*P* < 0.001 using one-way ANOVA with Tukey’s multiple comparison test. Bars indicate mean ± SEM.

To determine whether Duolac ATP has an effect on the balance of intestinal immune cells, mLN and PP were examined from AD mice treated with Duolac ATP. The proportion of DCs in the mLN and PP was slightly increased in AD mice given Duolac ATP (**Figure 6A**). Although the subpopulation of DCs did not change significantly, the number of CD11b^+^CD103^−^DCs that induce atopic inflammation decreased slightly after treatment with Duolac ATP in PP (**Supplementary Figure 6B**). Moreover, the number of CD4^+^ T cells in mLN and PP slightly decreased when Duolac ATP was administered (**Figure 6B**). It is well known that Tregs increase in response to a Th2-driven allergic reaction and that they are responsible for maintaining functional tolerance in an AD mouse model (Kim et al., 2013). Thus, we measured the ratio of Tregs in mLN and PP from AD mice with and without Duolac ATP treatment. Compared with PP, mLN had a relatively high proportion of Tregs but no change was found in any treatment group (**Figure 6C**). In, PP, however, Treg differentiation was increased in AD mice treated with Duolac ATP compared to control and untreated AD groups. We then examined the other subtypes of helper T cells by testing the expression IFN-gamma (Th1), IL-4 (Th2), and IL-17 (Th17) in CD4^+^ T cells after PMA/ionomycin stimulation. In the mLNs, only a small change (<1%) was observed in the subtype of CD4^+^ T cells in all groups (**Figure 6E**). Similar to mLN, there was little difference among Th1, Th2, and Th17 cells in PP across all treatment groups (**Figure 6F**). These results suggest that Duolac ATP is able to alleviate the symptoms of atopic disease by increasing the population of intestinal Treg cells.

## Discussion

Atopic dermatitis is a major allergic disease that results in increased IgE levels and clinically manifests itself as pruritic skin lesions, dermal hyperkeratosis, and hyperplasia (Abramovits, 2005; Silverberg, 2016). Recent studies have shown that certain probiotics can play a role in the treatment and prevention of AD (Kim et al., 2012, 2015). Duolac ATP possesses anti-inflammatory properties in a DNCB-induced AD mouse model (Kim et al., 2016). However, the exact mechanism by which Duolac ATP regulates the immune system has not been well defined. In the present study, we demonstrated the regulatory role of Duolac ATP in BMDCs and confirmed the mechanism by which transcription factors and cytokines have a protective effect against AD in mice.

As expected, mice treated with HDM- or DNCB-induced AD showed an excessive Th2 response in the present study. A number of different strategies have been suggested in the management of AD, including the preferential differentiation of T cells toward a Th1 lineage/blocking Th2 action. The T cell response is critically regulated by major transcription factors for both Th1 (T-bet, STAT1, STAT4) and Th2 (GATA-3, C-maf) responses (Rengarajan et al., 2000; Erpenbeck et al., 2006). IL-12 produced by antigen-presenting cells induces phosphorylation of STAT4, resulting in the subsequent production of IL-2 and IFN-gamma by Th1 cells (Frucht et al., 2001; Park et al., 2004). IFN-gamma then induces the activation of T-bet and STAT1, which, in turn, generate a positive feedback loop of the Th1 response (Ramana et al., 2002). Increased circulating T-bet concurrently inhibits the binding and activation of GATA-3 to the IL-4 gene promoter along with other Th2-related gene promoter (Kanhere et al., 2012).

In one study, PBMCs were taken from allergic patients and treated with various probiotics, including *L. plantarum*, *L. lactis*, and *L. casei*, resulting in reduced IL-4 secretion (Toh et al., 2012). Treatment with *L. plantarum*, one of the components of Duolac ATP, decreased T-bet and GATA-3 expression in the mouse small intestinal lamina propria (Smelt et al., 2013). T-bet expression also decreased in human PBMCs when stimulated with a single-strain probiotic *B. lactis* (Holvoet et al., 2013). A probiotic mixture called WIKIM28 decreased IL-4 and IL-5 levels, but not IFN-gamma, in peripheral lymph node cells (Lim et al., 2017). Administration of IRT5, another probiotic mixture, also reduced the levels of IL-4, IFN-gamma and the TNF-alpha in mesenteric lymph nodes (Kwon et al., 2010). Our results demonstrated that Duolac ATP induced upregulation of phospho-STAT1 and T-bet while downregulating GATA-3 in an AD mouse model. Furthermore, Duolac ATP increased IFN-gamma and decreased IL-4 *in vivo*. These results suggest that the use of multiple probiotic strains can lead to different Th1 responses.

Another strategy for overcoming AD is to suppress the overall immune response through regulatory cytokines, such as IL-10 and TGF-beta. These cytokines downregulate the activation of immune responses and therefore minimize the amount of tissue damage caused by inflammation in AD (Ng et al., 2009). IL-10 is produced by a number of immune cells, including DCs, macrophages, monocytes, T cells, and B cells (de Moreno de Leblanc et al., 2011). Microbial products stimulate pattern recognition receptors to increase the expression of mitogen-activated protein kinase kinase in DCs or macrophages, thereby increasing IL-10 production (Jang et al., 2004).

*Lactobacillus casei* and *L. rhamnosus* induce IL-10 production in allergic patients by regulating the composition of intestinal microbial flora (D’Inca et al., 2011). IL-10 production in Th2 cells was suppressed by IL-4 and GATA3 (Zhu et al., 2004; Mitchell et al., 2017). Given that Duolac ATP decreased IL-4 and GATA3 expression while increasing STAT4 levels, it is unlikely that IL-10 was secreted in Th2 cells. On the other hand, Duolac ATP induced upregulation of IL-10 in BMDCs and AD mice, suggesting that Duolac ATP may drive IL-10 production from cells other than Th2 cells, such as DCs.

TGF-beta is produced by many immune cells, and the TGF-beta signal is an essential component in maintaining healthy immune tolerance (Li et al., 2006). It has been suggested that TGF-beta can reduce allergic inflammation by inhibiting IgE synthesis in B-cells and mast cell proliferation (Gomez et al., 2005). TGF-beta is involved in the inhibitory action of *Lactobacillus acidophilus* on *Salmonella typhimurium*-induced inflammation (Huang et al., 2015). A mixture of probiotics, known as VSL#3, induced TGF-beta and alleviated allergic inflammation by reducing the Th2 response in a mouse model (Barletta et al., 2013). Similarly, we found that TGF-beta was increased when BMDCs were treated with Duolac ATP. TGF-beta secreted from DCs may regulate the differentiation of Tregs (Park et al., 2018). We confirmed that TGF-beta expression, which is slightly decreased in atopy, recovered to a normal level in mice after Duolac ATP treatment (**Supplementary Figure S5B**). Moreover, Duolac ATP promoted Treg differentiation in PPs (**Figure 5F**). It is likely that Duolac ATP modulates immune cells in a manner similar to how tolerogenic DCs maintain immune tolerance.

Immune imbalances caused by atopic reactions induce epidermal inflammation and hyperplasia (Abramovits, 2005; Jin et al., 2009). It has been suggested that oral administration of *Enterococcus faecalis* downregulates the production of Th1 and Th2 cytokines to attenuate skin hyperplasia (Choi et al., 2016). In the present study, Duolac ATP reduced IgE production by decreasing Th2 and increasing the Th1 response, thereby preventing skin inflammation and hyperplasia.

In summary, our data show that Duolac ATP regulated IL-10 and TGF-beta expression and allowed DCs to become functionally tolerant and potentially induce Treg differentiation. Furthermore, we found that Duolac ATP regulated transcription factors and cytokines to drive naïve T cell differentiation toward Th1 lineages. Taken together, these results indicate that Duolac ATP shows great preventive potential in the management of AD symptoms and could serve as a future immunomodulatory agent for AD patients.

## Author Contributions

C-HY and IC designed and performed the experiments and wrote the manuscript. EC and KC provided supervision during the study and manuscript preparation. HK, RH, KY, NK, and JH performed the experiments and analyzed the data. All authors reviewed the manuscript.

## Conflict of Interest Statement

The authors declare that the research was conducted in the absence of any commercial or financial relationships that could be construed as a potential conflict of interest.

## Supplementary Material

FIGURE S1 Schematic diagram for HDM extract- and DNCB-induced mouse model of atopic dermatitis (AD)-like skin lesions and oral administration of Duolac ATP in NC/Nga mice. NC/Nga mice were divided into three groups: (1) normal control (Control), (2) atopic dermatitis (AD), and (3) atopic dermatitis + Duolac ATP AD + Duolac ATP). To study the effect of Duolac ATP on atopic dermatitis, the dorsal skin and ears of the mice were treated with HDM extracts or DNCB six times over 3 weeks to induce AD-like skin lesions. Duolac ATP was administered following the last treatment with HDM extracts at week 0. At week 4, the mice were sacrificed, and the blood, spleen, PP, and mLN were collected for immunological analysis and dorsal skin histological analysis.

FIGURE S2 Apoptosis of BMDCs treated with probiotics that comprise Duolac ATP. BMDCs were treated with various concentrations of probiotics [Duolac ATP, *Bifidobacterium lactis* (BL), *Lactobacillus casei* (LC), *Lactobacillus plantarum* (LP), or *Lactobacillus rhamnosus* (LR)] for 24 h. The percentage of apoptotic cells, Shown as the annexin V-positive fraction, was measured. The concentration of BMDCs was 2 × 10^5^ cells in all groups. Data are representative of at least three experiments.

FIGURE S3 Expression of surface molecules on BMDCs treated with Duolac ATP. BMDCs were treated with LPS or 2 × 10^6^ CFU of Duolac ATP for 24 h. The ratios of CD86-, MHC II-, and PD-L1-expressing cells in BMDCs were measured by flow cytometry. Data are representative of at least three experiments. ^∗^*P* < 0.05, ^∗∗∗^*P* < 0.001 using one-way ANOVA with Tukey’s multiple comparison test. Bars indicate mean ± SEM.

FIGURE S4 Body and spleen weight changes in the AD mouse model treated with Duolac ATP. Atopic dermatitis was induced by exposing mice to HDM extracts for 3 weeks; mice were then administered Duolac ATP for 4 weeks. **(A)** Body weight was monitored weekly for 4 weeks. **(B)** The spleen was isolated and weighted at week 4.

FIGURE S5 Cytokine expression changes in the AD mouse treated with Duolac ATP. NC/Nga mice were sensitized by exposure to DNCB twice a week for 3 weeks. They were then orally administered with PBS or Duolac ATP for 4 weeks. Blood samples were taken and serum **(A)** IFN-gamma and **(B)** TGF-beta levels were measured by ELISA. Data are representative of at least three experiments. ^∗^*P* < 0.05 using one-way ANOVA with Tukey’s multiple comparison test. Bars indicate mean ± SEM.

FIGURE S6 Subpopulation of DC from mLN and PP in the AD mouse treated with Duolac ATP. NC/Nga mice were sensitized by exposing DNCB twice a week for 3 weeks. The mice were then orally administered PBS or Duolac ATP for 4 weeks. **(A)** mLN and **(B)** PP collected at week 4 were used to make single cells. The cells were gated on CD11c and subdivided based on CD11b and CD103 expression using flow cytometry. Data are representative of at least three experiments.

## References

[B1] Abramovits W. (2005). Atopic dermatitis. *J. Am. Acad. Dermatol.* 53 S86–S93. 10.1016/j.jaad.2005.04.034 15968268

[B2] Banchereau J. Steinman R. M. (1998). Dendritic cells and the control of immunity. *Nature* 392 245–252. 10.1038/32588 9521319

[B3] Barletta B. Rossi G. Schiavi E. Butteroni C. Corinti S. Boirivant M. (2013). Probiotic VSL#3-induced TGF-beta ameliorates food allergy inflammation in a mouse model of peanut sensitization through the induction of regulatory T cells in the gut mucosa. *Mol. Nutr. Food Res.* 57 2233–2244. 10.1002/mnfr.201300028 23943347

[B4] Cha Y. S. Seo J. G. Chung M. J. Cho C. W. Youn H. J. (2014). A mixed formulation of lactic acid bacteria inhibits trinitrobenzene-sulfonic-acid-induced inflammatory changes of the colon tissue in mice. *J. Microbiol. Biotechnol.* 24 1438–1444. 10.4014/jmb.1403.03064 24912557

[B5] Choi E. J. Iwasa M. Han K. I. Kim W. J. Tang Y. Hwang Y. J. (2016). Heat-killed *Enterococcus faecalis* EF-2001 ameliorates atopic dermatitis in a murine model. *Nutrients* 8:146. 10.3390/nu8030146 26959058 PMC4808875

[B6] Christensen H. R. Frokiaer H. Pestka J. J. (2002). Lactobacilli differentially modulate expression of cytokines and maturation surface markers in murine dendritic cells. *J. Immunol.* 168 171–178. 10.4049/jimmunol.168.1.171 11751960

[B7] Cuello-Garcia C. A. Brozek J. L. Fiocchi A. Pawankar R. Yepes-Nunez J. J. Terracciano L. (2015). Probiotics for the prevention of allergy: a systematic review and meta-analysis of randomized controlled trials. *J. Allergy Clin. Immunol.* 136 952–961. 10.1016/j.jaci.2015.04.031 26044853

[B8] de Moreno de Leblanc A. Del Carmen S. Zurita-Turk M. Santos Rocha C. Van De Guchte M. Azevedo V. (2011). Importance of IL-10 modulation by probiotic microorganisms in gastrointestinal inflammatory diseases. *ISRN Gastroenterol.* 2011:892971. 10.5402/2011/892971 21991534 PMC3168568

[B9] D’Inca R. Barollo M. Scarpa M. Grillo A. R. Brun P. Vettorato M. G. (2011). Rectal administration of *Lactobacillus casei* DG Modifies flora composition and toll-like receptor expression in colonic mucosa of patients with mild ulcerative colitis. *Dig. Dis. Sci.* 56 1178–1187. 10.1007/s10620-010-1384-1 20737210

[B10] Drakes M. Blanchard T. Czinn S. (2004). Bacterial probiotic modulation of dendritic cells. *Infect. Immun.* 72 3299–3309. 10.1128/IAI.72.6.3299-3309.2004 15155633 PMC415669

[B11] Erpenbeck V. J. Hagenberg A. Krentel H. Discher M. Braun A. Hohlfeld J. M. (2006). Regulation of GATA-3, c-maf and T-bet mRNA expression in bronchoalveolar lavage cells and bronchial biopsies after segmental allergen challenge. *Int. Arch. Allergy Immunol.* 139 306–316. 10.1159/000091602 16498264

[B12] Frucht D. M. Fukao T. Bogdan C. Schindler H. O’shea J. J. Koyasu S. (2001). IFN-gamma production by antigen-presenting cells: mechanisms emerge. *Trends Immunol.* 22 556–560. 10.1016/S1471-4906(01)02005-1 11574279

[B13] Gomez G. Ramirez C. D. Rivera J. Patel M. Norozian F. Wright H. V. (2005). TGF-beta 1 inhibits mast cell Fc epsilon RI expression. *J. Immunol.* 174 5987–5993. 10.4049/jimmunol.174.10.5987 15879091 PMC1391973

[B14] Gupta A. K. Chow M. (2003). Pimecrolimus: a review. *J. Eur. Acad. Dermatol. Venereol.* 17 493–503. 10.1046/j.1468-3083.2003.00692.x 12941081

[B15] Hart A. L. Lammers K. Brigidi P. Vitali B. Rizzello F. Gionchetti P. (2004). Modulation of human dendritic cell phenotype and function by probiotic bacteria. *Gut* 53 1602–1609. 10.1136/gut.2003.037325 15479680 PMC1774301

[B16] Hengge U. R. Ruzicka T. Schwartz R. A. Cork M. J. (2006). Adverse effects of topical glucocorticosteroids. *J. Am. Acad. Dermatol.* 54 1–15; quiz 16–18. 10.1016/j.jaad.2005.01.010 16384751

[B17] Holvoet S. Zuercher A. W. Julien-Javaux F. Perrot M. Mercenier A. (2013). Characterization of candidate anti-allergic probiotic strains in a model of Th2-skewed human peripheral blood mononuclear cells. *Int. Arch. Allergy Immunol.* 161 142–154. 10.1159/000343703 23343780

[B18] Hong S. W. Kim K. S. Surh C. D. (2017). Beyond hygiene: commensal microbiota and allergic diseases. *Immune Netw.* 17 48–59. 10.4110/in.2017.17.1.48 28261020 PMC5334122

[B19] Huang I. F. Lin I. C. Liu P. F. Cheng M. F. Liu Y. C. Hsieh Y. D. (2015). *Lactobacillus acidophilus* attenuates *Salmonella*-induced intestinal inflammation via TGF-beta signaling. *BMC Microbiol.* 15:203. 10.1186/s12866-015-0546-x 26446848 PMC4596496

[B20] Isolauri E. (2001). Probiotics in human disease. *Am. J. Clin. Nutr.* 73 1142S–1146S. 10.1093/ajcn/73.6.1142S 11393192

[B21] Isolauri E. Arvola T. Sutas Y. Moilanen E. Salminen S. (2000). Probiotics in the management of atopic eczema. *Clin. Exp. Allergy* 30 1604–1610. 10.1046/j.1365-2222.2000.00943.x 11069570

[B22] Jang S. Uematsu S. Akira S. Salgame P. (2004). IL-6 and IL-10 induction from dendritic cells in response to *Mycobacterium tuberculosis* is predominantly dependent on TLR2-mediated recognition. *J. Immunol.* 173 3392–3397. 10.4049/jimmunol.173.5.3392 15322203

[B23] Jin H. He R. Oyoshi M. Geha R. S. (2009). Animal models of atopic dermatitis. *J. Invest. Dermatol.* 129 31–40. 10.1038/jid.2008.106 19078986 PMC2886143

[B24] Kanhere A. Hertweck A. Bhatia U. Gokmen M. R. Perucha E. Jackson I. (2012). T-bet and GATA3 orchestrate Th1 and Th2 differentiation through lineage-specific targeting of distal regulatory elements. *Nat. Commun.* 3:1268. 10.1038/ncomms2260 23232398 PMC3535338

[B25] Kim J. Y. Park B. K. Park H. J. Park Y. H. Kim B. O. Pyo S. (2013). Atopic dermatitis-mitigating effects of new *Lactobacillus* strain. *J. Appl. Microbiol.* 115 517–526. 10.1111/jam.12229 23607518

[B26] Kim M. S. Kim J. E. Yoon Y. S. Kim T. Seo J. G. Chung M. J. (2015). Improvement of atopic dermatitis-like skin lesions by IL-4 inhibition of P14 protein isolated from *Lactobacillus casei* in NC/Nga mice. *Appl. Microbiol. Biotechnol.* 99 7089–7099. 10.1007/s00253-015-6455-y 25687448

[B27] Kim M. S. Kim J. E. Yoon Y. S. Seo J. G. Chung M. J. Yum D. Y. (2016). A probiotic preparation alleviates atopic dermatitis-like skin lesions in murine models. *Toxicol. Res.* 32 149–158. 10.5487/TR.2016.32.2.149 27123166 PMC4843972

[B28] Kim M. S. Kim W. G. Chung H. S. Park B. W. Ahn K. S. Kim J. J. (2012). Improvement of atopic dermatitis-like skin lesions by *Platycodon grandiflorum* fermented by *Lactobacillus plantarum* in NC/Nga Mice. *Biol. Pharm. Bull.* 35 1222–1229. 10.1248/bpb.b110504 22863917

[B29] Kwon H. K. Lee C. G. So J. S. Chae C. S. Hwang J. S. Sahoo A. (2010). Generation of regulatory dendritic cells and CD4 + Foxp3 + T cells by probiotics administration suppresses immune disorders. *Proc. Natl. Acad. Sci. U.S.A.* 107 2159–2164. 10.1073/pnas.0904055107 20080669 PMC2836639

[B30] Lavasani S. Dzhambazov B. Nouri M. Fak F. Buske S. Molin G. (2010). A novel probiotic mixture exerts a therapeutic effect on experimental autoimmune encephalomyelitis mediated by IL-10 producing regulatory T cells. *PLoS One* 5:e9009. 10.1371/journal.pone.0009009 20126401 PMC2814855

[B31] Lee H. K. Iwasaki A. (2007). Innate control of adaptive immunity: dendritic cells and beyond. *Semin. Immunol.* 19 48–55. 10.1016/j.smim.2006.12.001 17276695

[B32] Li M. O. Wan Y. Y. Sanjabi S. Robertson A. K. L. Flavell R. A. (2006). Transforming growth factor-beta regulation of immune responses. *Annu. Rev. Immunol.* 24 99–146. 10.1146/annurev.immunol.24.021605.090737 16551245

[B33] Lim S. K. Kwon M. S. Lee J. Oh Y. J. Jang J. Y. Lee J. H. (2017). *Weissella* cibaria WIKIM28 ameliorates atopic dermatitis-like skin lesions by inducing tolerogenic dendritic cells and regulatory T cells in BALB/c mice. *Sci. Rep.* 7:40040. 10.1038/srep40040 28067304 PMC5220369

[B34] Lindfors K. Blomqvist T. Juuti-Uusitalo K. Stenman S. Venalainen J. Maki M. (2008). Live probiotic *Bifidobacterium* lactis bacteria inhibit the toxic effects induced by wheat gliadin in epithelial cell culture. *Clin. Exp. Immunol.* 152 552–558. 10.1111/j.1365-2249.2008.03635.x 18422736 PMC2453197

[B35] Mainardi T. Kapoor S. Bielory L. (2009). Complementary and alternative medicine: herbs, phytochemicals and vitamins and their immunologic effects. *J. Allergy Clin. Immunol.* 123 283–294; quiz 295–296. 10.1016/j.jaci.2008.12.023 19203652

[B36] Mercenier A. Pavan S. Pot B. (2003). Probiotics as biotherapeutic agents: present knowledge and future prospects. *Curr. Pharm. Des.* 9 175–191. 10.2174/1381612033392224 12570667

[B37] Mitchell R. E. Hassan M. Burton B. R. Britton G. Hill E. V. Verhagen J. (2017). IL-4 enhances IL-10 production in Th1 cells: implications for Th1 and Th2 regulation. *Sci. Rep.* 7:11315. 10.1038/s41598-017-11803-y 28900244 PMC5595963

[B38] Ng S. C. Hart A. L. Kamm M. A. Stagg A. J. Knight S. C. (2009). Mechanisms of action of probiotics: recent advances. *Inflamm. Bowel Dis.* 15 300–310. 10.1002/ibd.20602 18626975

[B39] Park H. J. Lee S. W. Hong S. (2018). Regulation of allergic immune responses by microbial metabolites. *Immune Netw.* 18:e15. 10.4110/in.2018.18.e15 29503745 PMC5833122

[B40] Park W. R. Nakahira M. Sugimoto N. Bian Y. Yashiro-Ohtani Y. Zhou X. Y. (2004). A mechanism underlying STAT4-mediated up-regulation of IFN-gamma induction inTCR-triggered T cells. *Int. Immunol.* 16 295–302. 10.1093/intimm/dxh034 14734615

[B41] Powrie F. (2004). Immune regulation in the intestine: a balancing act between effector and regulatory T cell responses. *Ann. N. Y. Acad. Sci.* 1029 132–141. 10.1196/annals.1309.030 15681752

[B42] Prakoeswa C. R. S. Herwanto N. Prameswari R. Astari L. Sawitri S. Hidayati A. N. (2017). *Lactobacillus* plantarum IS-10506 supplementation reduced SCORAD in children with atopic dermatitis. *Benef. Microbes* 8 833–840. 10.3920/BM2017.0011 29022387

[B43] Ramana C. V. Gil M. P. Schreiber R. D. Stark G. R. (2002). Stat1-dependent and -independent pathways in IFN-gamma-dependent signaling. *Trends Immunol.* 23 96–101. 10.1016/S1471-4906(01)02118-4 11929133

[B44] Rather I. A. Bajpai V. K. Kumar S. Lim J. Paek W. K. Park Y. H. (2016). Probiotics and atopic dermatitis: an overview. *Front. Microbiol.* 7:507 10.3389/fmicb.2016.00507 PMC4828648 27148196

[B45] Reid G. Sanders M. E. Gaskins H. R. Gibson G. R. Mercenier A. Rastall R. (2003). New scientific paradigms for probiotics and prebiotics. *J. Clin. Gastroenterol.* 37 105–118. 10.1097/00004836-200308000-00004 12869879

[B46] Rengarajan J. Szabo S. J. Glimcher L. H. (2000). Transcriptional regulation of Th1/Th2 polarization. *Immunol. Today* 21 479–483. 10.1016/S0167-5699(00)01712-6 11071525

[B47] Shin J. H. Chung M. J. Seo J. G. (2016). A multistrain probiotic formulation attenuates skin symptoms of atopic dermatitis in a mouse model through the generation of CD4( + )Foxp3( + ) T cells. *Food Nutr. Res.* 60:32550. 10.3402/fnr.v60.32550 27802847 PMC5090133

[B48] Silverberg N. B. (2016). A practical overview of pediatric atopic dermatitis. *Cutis* 97 267–271. 27163911

[B49] Smelt M. J. De Haan B. J. Bron P. A. Van Swam I. Meijerink M. Wells J. M. (2013). Probiotics can generate foxP3 T-cell responses in the small intestine and simultaneously inducing CD4 and CD8 T cell activation in the large intestine. *PLoS One* 8:e68952. 10.1371/journal.pone.0068952 23861953 PMC3701681

[B50] Tanaka A. Jung K. Benyacoub J. Prioult G. Okamoto N. Ohmori K. (2009). Oral supplementation with *Lactobacillus* rhamnosus CGMCC 1.3724 prevents development of atopic dermatitis in NC/NgaTnd mice possibly by modulating local production of IFN-gamma. *Exp. Dermatol.* 18 1022–1027. 10.1111/j.1600-0625.2009.00895.x 19555432

[B51] Toh Z. Q. Anzela A. Tang M. L. K. Licciardi P. V. (2012). Probiotic therapy as a novel approach for allergic disease. *Front. Pharmacol.* 3:171. 10.3389/fphar.2012.00171 23049509 PMC3448073

[B52] Woodfolk J. A. (2007). T-cell responses to allergens. *J. Allergy Clin. Immunol.* 119 280–294; quiz 295–296. 10.1016/j.jaci.2006.11.008 17291848

[B53] Zhu J. F. Min B. Hu-Li J. Watson C. J. Grinberg A. Wang Q. (2004). Conditional deletion of Gata3 shows its essential function in T(H)1-T(H)2 responses. *Nat. Immunol.* 5 1157–1165. 10.1038/ni1128 15475959

