# Supplementary figures and images for: A Probiotic Mixture Regulates T Cell Balance and Reduces Atopic Dermatitis Symptoms in Mice
Source: Front Microbiol. 2018 Oct 15;9:2414. doi: 10.3389/fmicb.2018.02414 (PMC6196311; doi:10.3389/fmicb.2018.02414)

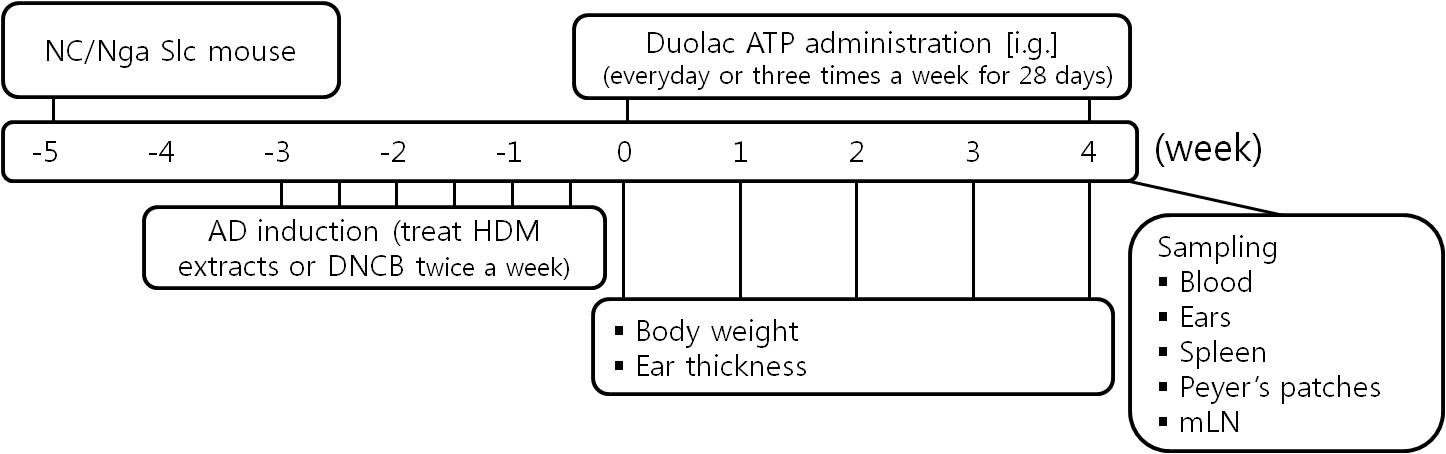

Supplement: FIGURE S1 — Schematic diagram for HDM extract- and DNCB-induced mouse model of atopic dermatitis (AD)-like skin lesions and oral administration of Duolac ATP in NC/Nga mice. NC/Nga mice were divided into three groups: (1) normal control (Control), (2) atopic dermatitis (AD), and (3) atopic dermatitis + Duolac ATP AD + Duolac ATP). To study the effect of Duolac ATP on atopic dermatitis, the dorsal skin and ears of the mice were treated with HDM extracts or DNCB six times over 3 weeks to induce AD-like skin lesions. Duolac ATP was administered following the last treatment with HDM extracts at week 0. At week 4, the mice were sacrificed, and the blood, spleen, PP, and mLN were collected for immunological analysis and dorsal skin histological analysis. [file Image_1.jpg]

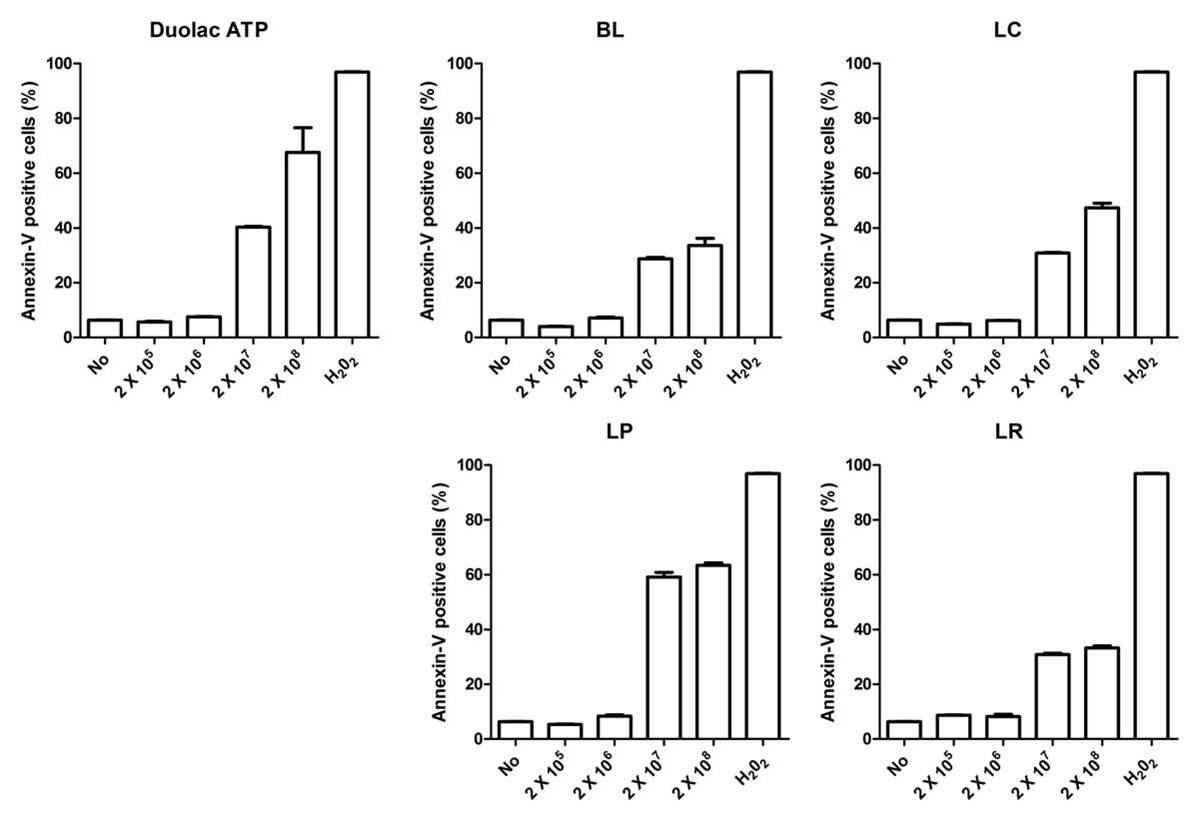

Supplement: FIGURE S2 — Apoptosis of BMDCs treated with probiotics that comprise Duolac ATP. BMDCs were treated with various concentrations of probiotics [Duolac ATP, Bifidobacterium lactis (BL), Lactobacillus casei (LC), Lactobacillus plantarum (LP), or Lactobacillus rhamnosus (LR)] for 24 h. The percentage of apoptotic cells, Shown as the annexin V-positive fraction, was measured. The concentration of BMDCs was 2 × 10^5^ cells in all groups. Data are representative of at least three experiments. [file Image_2.jpg]

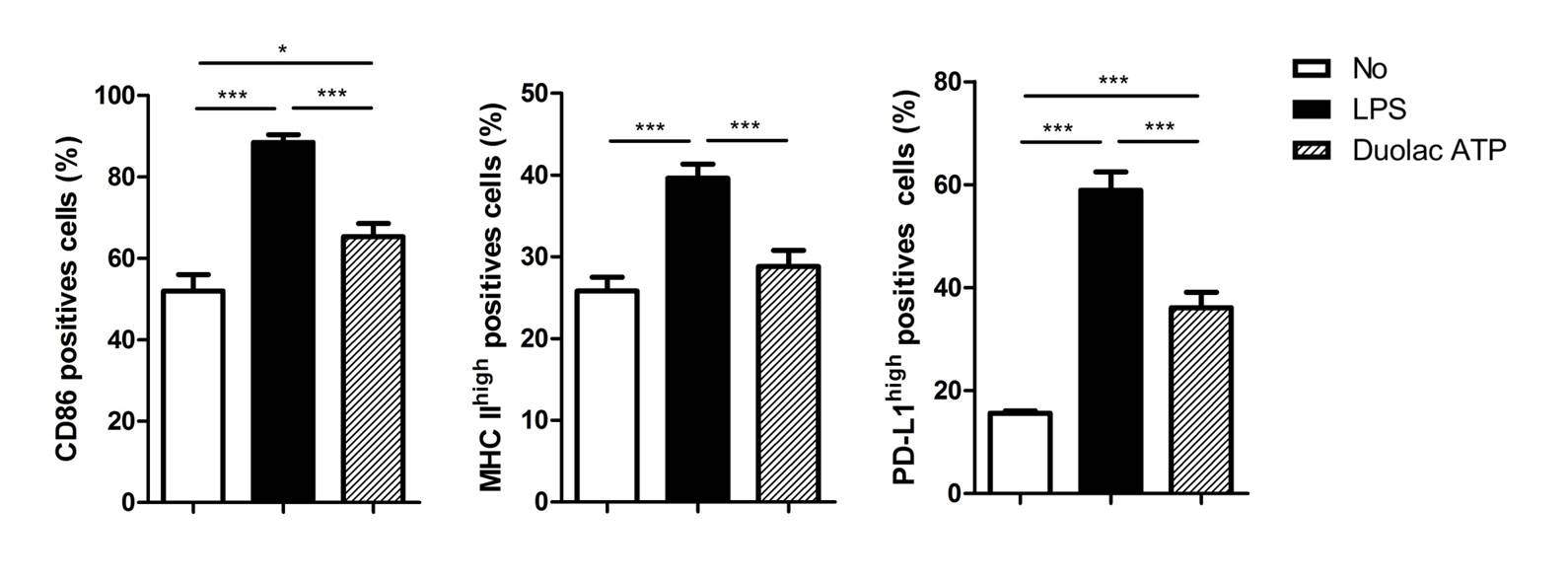

Supplement: FIGURE S3 — Expression of surface molecules on BMDCs treated with Duolac ATP. BMDCs were treated with LPS or 2 × 10^6^ CFU of Duolac ATP for 24 h. The ratios of CD86-, MHC II-, and PD-L1-expressing cells in BMDCs were measured by flow cytometry. Data are representative of at least three experiments. ∗P < 0.05, ∗∗∗P < 0.001 using one-way ANOVA with Tukey’s multiple comparison test. Bars indicate mean ± SEM. [file Image_3.jpg]

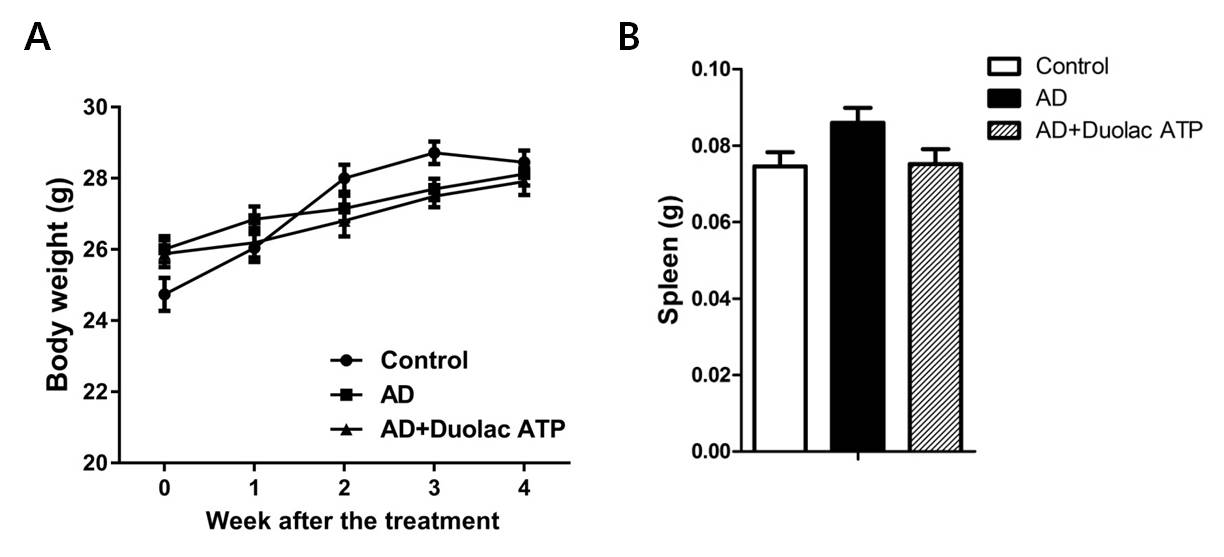

Supplement: FIGURE S4 — Body and spleen weight changes in the AD mouse model treated with Duolac ATP. Atopic dermatitis was induced by exposing mice to HDM extracts for 3 weeks; mice were then administered Duolac ATP for 4 weeks. (A) Body weight was monitored weekly for 4 weeks. (B) The spleen was isolated and weighted at week 4. [file Image_4.JPEG]

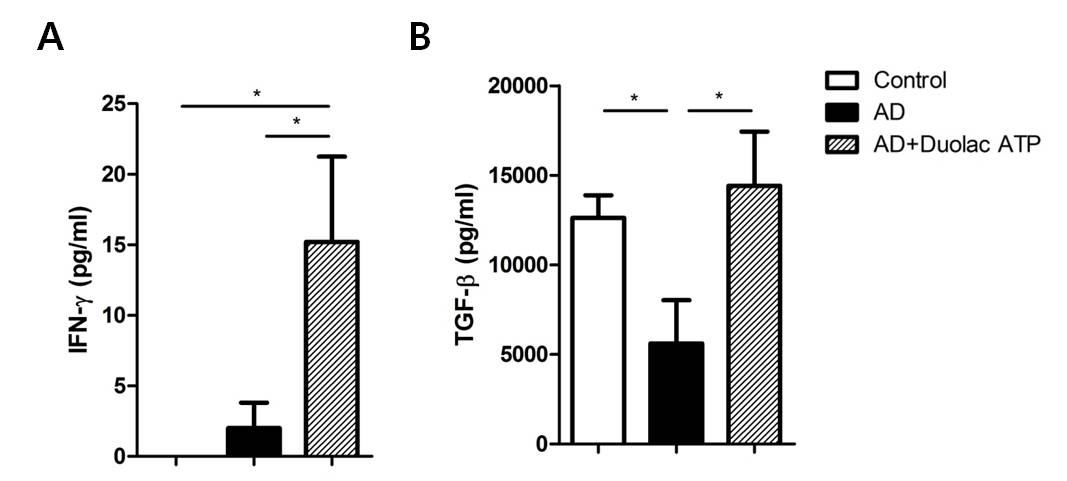

Supplement: FIGURE S5 — Cytokine expression changes in the AD mouse treated with Duolac ATP. NC/Nga mice were sensitized by exposure to DNCB twice a week for 3 weeks. They were then orally administered with PBS or Duolac ATP for 4 weeks. Blood samples were taken and serum (A) IFN-gamma and (B) TGF-beta levels were measured by ELISA. Data are representative of at least three experiments. ∗P < 0.05 using one-way ANOVA with Tukey’s multiple comparison test. Bars indicate mean ± SEM. [file Image_5.jpg]

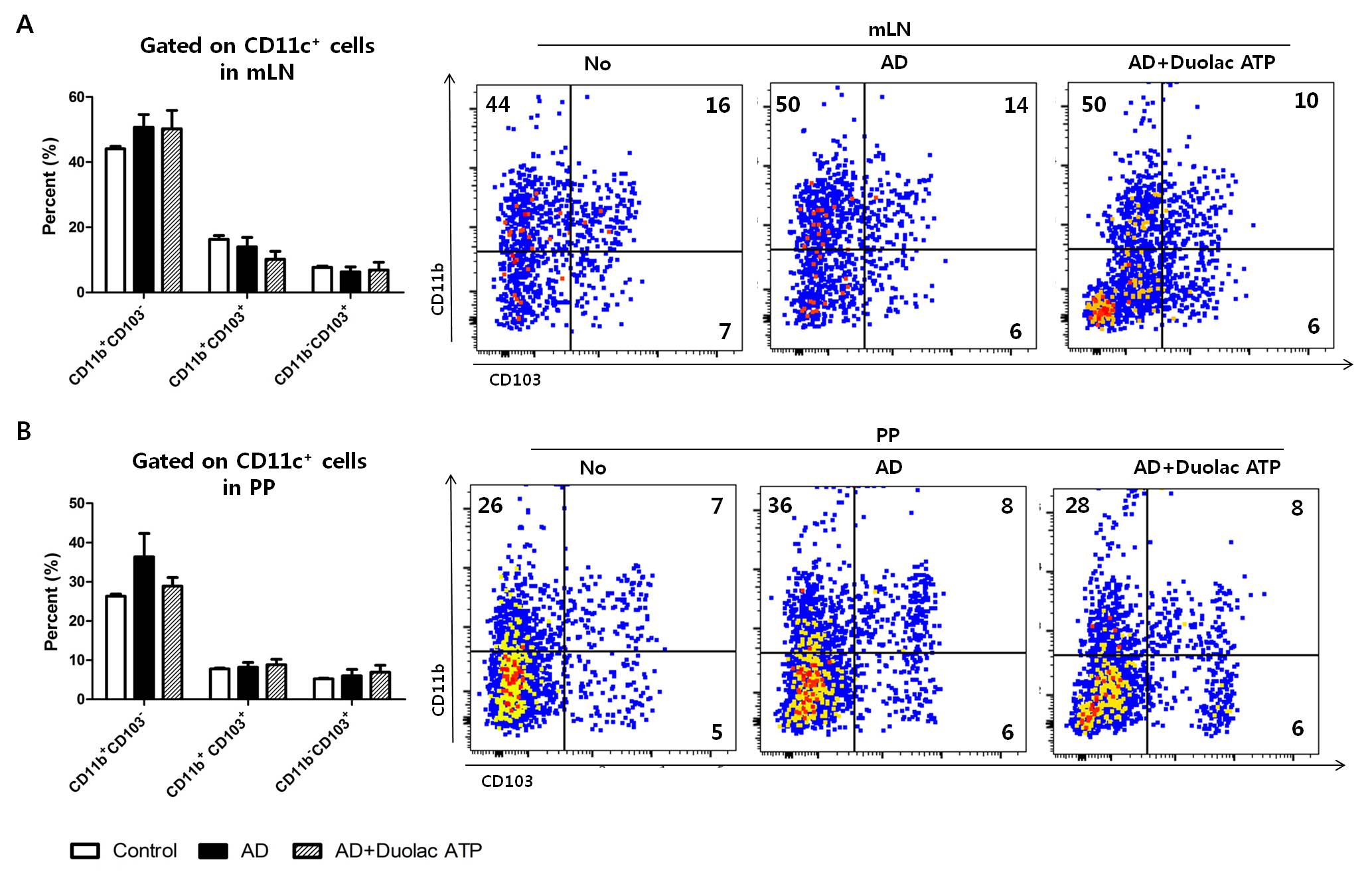

Supplement: FIGURE S6 — Subpopulation of DC from mLN and PP in the AD mouse treated with Duolac ATP. NC/Nga mice were sensitized by exposing DNCB twice a week for 3 weeks. The mice were then orally administered PBS or Duolac ATP for 4 weeks. (A) mLN and (B) PP collected at week 4 were used to make single cells. The cells were gated on CD11c and subdivided based on CD11b and CD103 expression using flow cytometry. Data are representative of at least three experiments. [file Image_6.jpg]
